# Supplementary material for: Genome-wide transcriptome analysis identifies alternative splicing regulatory network and key splicing factors in mouse and human psoriasis
Source: Sci Rep. 2018 Mar 7;8:4124. doi: 10.1038/s41598-018-22284-y (PMC5841439; doi:10.1038/s41598-018-22284-y)
Supplement: Supplementary file 1 — Supplements [file 41598_2018_22284_MOESM1_ESM.docx]

Peng Yu, PhD

Department of Electrical and Computer Engineering & TEES-AgriLife Center for Bioinformatics and Genomic Systems Engineering,

Texas A&M University,

College Station, TX 77843, USA

Tel: 1-979-845-7441

Fax: 1-979-845-6259

Email: [pengyu.bio@gmail.com](mailto:lzheng.chn@gmail.com)

**Genome-wide transcriptome analysis identifies alternative splicing regulatory network and key splicing factors in mouse and human psoriasis**

Jin Li^1,2^ and Peng Yu^1,2,*^

^1^Department of Electrical and Computer Engineering & ^2^TEES-AgriLife Center for Bioinformatics and Genomic Systems Engineering, Texas A&M University, College Station, TX 77843, USA

*To whom correspondence should be addressed.

**Running title:** Alternative splicing in psoriasis

**Keyword:** Alternative splicing; Splicing factor; Regulatory network; Psoriasis

**Supplemental Document S1. A representative multiple sequence alignment of the sequences of the splicing event of *Exoc1/EXOC1* in mice and humans.** To investigate the conservation of splicing isoform sequences, we extracted two isoform sequences that cover the differential exon skipping events in *Exoc1* from mice*.* We also extracted two isoform sequences that cover the differential exon skipping events in *EXOC1* from humans*.* MAFFT was used to construct the multiple sequence alignment (MSA) of the four sequences. From the MSA of *Exoc1/EXOC1* isoform sequences, we were able to identify the conserved variable exon and the conserved upstream and downstream exons.

**Figure S1. UCSC genome browser tracks visualization for the DAS events in *Fnbp1/FNBP1* and *Atp5c1/ATP5C1*.** (a) The visualization of the DAS event in *Fnbp1/FNBP1*. The psoriatic samples have less inclusion of variable exons in both mice and humans. (b) The visualization of the DAS event in *Atp5c1/ATP5C1*. The psoriatic samples have more inclusion of variable exons in both mice and humans.

**Figure S2. GO terms identified for the genes with DAS events in the *Tnip1* KO mice and the human psoriasis dataset.** To examine the biological functions of the genes that have DAS events, gene ontology analysis was applied to identify enriched GO terms using the Fisher’s exact test. The figure depicts the enriched GO terms in red bars (log10 *p-*value) and green bars (log10 odds ratio). (a) Enriched GO terms for the genes with DAS events in the *Tnip1* KO mice. (b) Enriched GO terms for the genes with DAS events in the human psoriasis dataset.

**Figure S3. GO terms identified for the up-regulated genes in the *Tnip1* KO mice and the human psoriasis dataset.** To examine the biological functions of up-regulated genes, gene ontology analysis was performed. The figure depicts the enriched GO terms in red bars (log10 *p-*value) and green bars (log10 odds ratio). (a) Enriched GO terms for up-regulated genes in the *Tnip1* KO mice. (b) Enriched GO terms for up-regulated genes in the human psoriasis dataset.

**SUPPLEMENTAL TABLE LEGENDS**

**Table S1.** Differential alternative splicing events in the *Tnip1* KO mice and the human psoriasis dataset.

**Table S2.** Enriched gene ontology terms of DAS events in the Tnip1 KO mice and the human psoriasis dataset.

**Table S3**. Identification of the splicing events with the conserved sequences between the *Tnip1* KO mouse model and the human psoriasis dataset using multiple sequence alignment analysis.

**Table S4**. Identification of the candidate splicing factors for regulating the alternative splicing events in psoriasis.

**Table S5**. The identification of the splicing factors that potentially regulate the conserved splicing events by comparing the candidate splicing regulation in the mouse/human dataset and the SF perturbation datasets.

**Table S6.** Enriched gene ontology terms of the differentially spliced genes in the *Tnip1* KO mice and the human psoriasis dataset.

**SUPPLEMENTAL DOCUMENT LEGENDS**

**Supplemental Data S1. The multiple sequence alignments of 24 exon skipping events with conserved isoform sequences in mice and humans.** For each homologous gene in mice and humans, the two isoform sequences that cover the exon skipping event in mouse or human were extracted, and a multiple sequence alignment was constructed using MAFFT. The isoform sequences with conserved in the variable exons and the upstream/downstream exons were saved. The corresponding UCSC genome browser tracks of these conserved events in mice and humans were extracted.
